# Increased expression of upstream TH_2_-cytokines in a mouse model of viral-induced asthma exacerbation

**DOI:** 10.1186/s12967-016-0808-x

**Published:** 2016-02-16

**Authors:** Irma Mahmutovic Persson, Hamid Akbarshahi, Mandy Menzel, Angelica Brandelius, Lena Uller

**Affiliations:** Department Experimental Medical Science, Unit of Respiratory Immunopharmacology, Lund University, BMC D12, 221 84 Lund, Sweden

**Keywords:** Asthma, Exacerbation, HDM, DAMP, IL-33, TSLP, IL-25, RIG-I-like receptors, TLR3

## Abstract

**Background:**

Exacerbations of asthma caused by respiratory viral infections are serious conditions in need of novel treatment. To this end animal models of asthma exacerbations are warranted. We have shown that dsRNA challenges or rhinoviral infection produce exacerbation effects in mice with ovalbumin (OVA)-induced allergic asthma. However, house dust mite (HDM) is a more human asthma-relevant allergen than OVA. We thus hypothesised that dsRNA challenges in mice with HDM-induced experimental asthma would produce important translational features of asthma exacerbations.

**Method:**

Mouse airways were challenged locally with HDM or saline three times a week for three weeks to establish experimental asthma. Then daily local dsRNA challenges were given for three consecutive days to induce exacerbation. Bronchoalveolar lavage fluid (BALF) was analysed for inflammatory cells, total protein, the necrosis marker LDH and the alarmin ATP. Lung homogenates were analysed for mRNA expression (RT-qPCR) of TNF-α, CCL2, CCL5, IL-1β, IL-33, thymic stromal lymphopoietin (TSLP), and IL-25 as well as pattern recognition receptors (PRRs) RIG-I, MDA5 and TLR3. Lung tissue IL-33 was analysed with ELISA and PRRs were quantified by western blot. Immunohistochemistry indicated lung distribution of IL-33.

**Results:**

HDM challenge alone caused sustained increase in BALF total protein, eosinophils, lymphocytes and neutrophils, and transient increase in lung tissue expression of TSLP, IL-33 and TNF-α. dsRNA-induced exacerbation markedly and dose-dependently exaggerated these effects. Further, BALF levels of LDH and ATP, and lung tissue expression of CCL2, CCL5, IL-1β, IL-25 and PRRs were increased exclusively at the exacerbations. Lung protein levels of IL-33 were transiently increased by HDM and further increased at exacerbation.

**Conclusion:**

We demonstrate several novel aspects of HDM-induced experimental asthma and added exacerbation effects of dsRNA. General inflammatory parameters in BALF such as exuded proteins, mixed granulocytes, LDH and ATP were increased at the present exacerbations as they are in human asthma exacerbations. We suggest that this model of asthma exacerbation involving dsRNA challenges given to mice with established HDM-induced asthma has translational value and suggest that it may be particularly suited for in vivo studies involving pharmacological effects on exacerbation-induced expression of major upstream TH_2_-cytokines; IL-33, TSLP and IL-25, as well as PRRs.

**Electronic supplementary material:**

The online version of this article (doi:10.1186/s12967-016-0808-x) contains supplementary material, which is available to authorized users.

## Background

Roles of the cytokines IL-33, thymic stromal lymphopoietin (TSLP) and IL-25 in asthma are receiving considerable interest [1]. These three factors are defined as upstream epithelial cytokines driving the production of potent TH_2_-cytokines such as IL-5 and IL-13, which have proven to be involved in severe, difficult-to-treat eosinophilic asthma [2]. Severe asthma includes exacerbations, most commonly caused by rhinovirus (RV) infections [3–5]. Asthma exacerbations are characterised by increased eosinophilic and neutrophilic inflammation with increased levels of numerous humoral and cellular inflammatory factors including the TH_2_-type cytokines [6–8]. Exacerbations are not only episodes of severe asthma but may also drive development of the disease. Hence, pathogenic and pharmacological aspects of viral-induced exacerbations are important fields of research. However, it is only recently that tentative mouse models of asthma exacerbations have been emerging [9, 10], and little is as yet known regarding expression of cytokines and innate immunity receptors at viral stimulus-induced exacerbations compared to a baseline of allergic lung inflammation.

Traditionally mouse models of asthma involve sensitisation and airway challenge with ovalbumin (OVA) to produce allergic lung inflammation. We recently demonstrated that RV infection, or local lung administration of a RV-like stimulus such as dsRNA, produced markedly increased lung inflammation in mice with already established OVA-induced experimental asthma [11]. Our developed scheme of dsRNA challenges to mimic exacerbation effects of rhinoviral infection was thus validated [11]. Recently it has been claimed that each of a variety of cytokines, including IL-4, IL-17A, and IL-25, are essential drivers of rhinoviral induced exacerbation effects in OVA mouse models of asthma [12–14]. Culprit cells including macrophages and NK cells have also been singled out [13, 14]. However, there is a controversy as to what extent current mouse models may mimic essential features of severe asthma [15, 16]. In this regard improved mouse models of asthma exacerbations seem warranted.

The advantage of OVA models is development of robust pulmonary eosinophilia. However, several features of the OVA mouse models of asthma have been criticised because the allergen, the sensitisation route and the need for an artificial adjuvant have little relevance to human asthma [9, 15, 17]. In the last few years house dust mite (HDM) has been increasingly employed as allergen in attempts at creating improved mouse models of human asthma [9, 15, 17]. HDM is an environmentally relevant allergen known to be a trigger of human asthma. Its advantages over OVA further include sensitisation through respiratory routes and no need for adjuvants. Only a few HDM mouse models have emerged where attempts at producing viral stimuli-evoked exacerbations have been made. Limited signs of exacerbation have been observed beyond a pulmonary neutrophilia on top of a neutrophilic effect of HDM exposure alone [12, 18]. One apparent drawback may have involved the HDM exposure-induced baseline where inconsistent effects have been produced including lack of pulmonary eosinophilia [18]. In addition, viral infection or exposure to dsRNA have produced limited increases in pulmonary cytokines and none of the upstream bronchial epithelial TH_2_-cytokines has so far been reported in viral-HDM exacerbation models [12]. Yet, clinical studies support a role of these cytokines in human asthma [1, 19].

We hypothesised that a mouse model of HDM-induced eosinophilic inflammation would exhibit increased expression of upstream cytokines and that this feature would be exaggerated at exacerbations. In this study we thus aimed to develop a translational mouse model of viral-induced asthma exacerbation using airway challenges with HDM combined with viral exacerbation evoked by dsRNA. We focused our analysis in part on lung expression of the three major upstream cytokines IL-33, TSLP and IL-25. We also determined pattern recognition receptors (PRRs) in the lung after HDM challenges as well as during exacerbation evoked by dsRNA. Here we demonstrate that viral-induced HDM-asthma exacerbations associate with increased lung expression of three upstream cytokines whereas IL-33 and TSLP were also induced by HDM alone although to a lesser extent. Interestingly, PRRs (RIG-I, MDA5 and TLR3) were not induced by HDM alone but were markedly induced at the present exacerbation. General inflammatory variables such as exuded proteins, mixed granulocytes, LDH and ATP were all significantly increased in bronchoalveolar lavage fluid (BALF) during exacerbation as they are in human severe asthma [20, 21]. We suggest that this novel model of viral-induced asthma exacerbation has translational value and is suited for in vivo studies involving pharmacological effects on exacerbation-induced expression of IL-33, TSLP and IL-25, as well as PRRs.

## Methods

### Animals and experimental study design

**Fig. 1:**
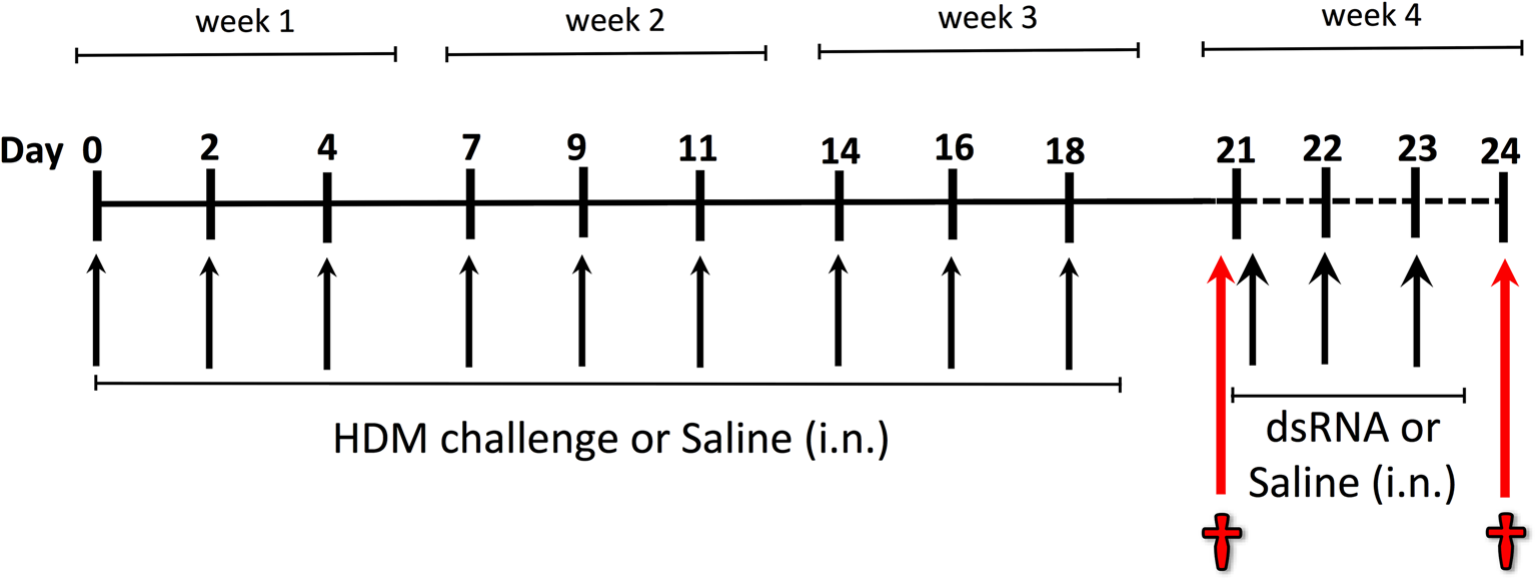
Study design involving dsRNA challenges to mice with established HDM-induced experimental asthma. Mice received three intranasal (i.n) challenges of HDM extract per week (or saline as control), during three weeks to establish allergic ‘asthma’. This was followed by an exacerbation phase achieved by additional i.n. administration of dsRNA (or saline as control) on three consecutive days (day 21–23). dsRNA was given either as low-dose (50 μg) or high-dose (100 μg) every 24 h. The mice were sacrificed, either 24 h after three weeks of HDM challenge or after the exacerbation phase, 24 h after the final dsRNA administration

Male mice (10 weeks old) with C57BL/6 background from Charles River were used in the study. The mice had free access to food and water during the whole period. The Animal Ethics Committee at Lund University approved all the experiments performed. Mice were challenged with HDM *Dermatophagoides pteronyssinus* whole extract (GREER, Lenoir, USA) dissolved in saline (to final concentration of 1 mg/ml) and given every other day during three weeks, while saline was administered as control. Mice were divided in six groups; Naïve (n = 4), Saline (n = 5), HDM (n = 6), HDM/Saline (n = 5), HDM/dsRNA50 (n = 6) and HDM/dsRNA100 (n = 6). The first three groups were sacrificed at day 21 (Naïve, Saline, HDM), to study the effects of HDM challenge alone. The experimental protocol of HDM-induced allergic airway inflammation was adapted from previous work done by Gregory et al. [22, 23]. Subsequently, to induce asthma exacerbation, the remaining three groups with HDM-challenged allergic background all received intranasal saline or dsRNA {polyinosine-polycytidylic acid [Poly(I:C)]; (Invitrogen Ltd, Paisley, UK)} as a rhinoviral mimic, administered as previously described in our laboratory [11]. Briefly, saline or dsRNA was administered on three consecutive days, using two different doses of dsRNA, a low-dose of dsRNA (50 μg) or a high-dose of dsRNA (100 μg). The experiment was terminated 24 h after the last intranasal administration, on day 24. The volume of all intranasal administrations was 25 μl. For experimental design see Fig. 1.

### Bronchoalveolar lavage and lung dissection

BALF was collected 24 h after the final intranasal administration as previously described [11]. The lungs were dissected and the left lung was fixed in 4 % formaldehyde while the right lung lobes were weighed and snap frozen in liquid nitrogen until homogenised for analysis with RT-qPCR, ELISA or western blot.

### Analysis of BALF

The BALF was centrifuged and the supernatant stored at −80 °C until further analysis. The cell pellet was re-suspended and total cell count was performed, using an automatic cell counter as previously described [11]. Subsequently, the cell suspension was cytospin-centrifuged onto microscope slides, stained and 400 cells per sample were differentially counted as previously described [11]. The cytospin slides were photographed and scored for epithelial cell count referred to as epithelial cell shedding score. Score was given depending on if there were any epithelial cells present in the sample. The number of clusters with epithelial cells were quantified and given a score 1–4 depending on the cluster size. Finally, the total number of epithelial cells in each sample was quantified and given a score (<10 cells = score 0; 11–25 cells = score 1; 26–50 cells = score 2; 51–100 cells = score 3 and >100 cells = score 4). The different scores were added together to obtain a total sample score.

The BALF supernatant was analysed for total protein concentration using bicinchoninic acid (BCA) assay (Pierce^®^ BCA Protein Assay Kit detection, Fischer Scientific AB, Sweden), according to the manufacturer’s instructions. Further, BALF was analysed with kit, for LDH (Roche Diagnostics, Sweden) and ATP levels (BioThema, Sweden) according to the manufacturer’s instructions.

### Analysis of lung homogenate

Frozen lung tissue was homogenised mechanically (OmniPrep Rotor Stator Generator, Omni International, USA) and enzymatically by addition of lysis buffer. Lung homogenate samples prepared for ELISA were lysed with lysis buffer with protease inhibitors (Roche Diagnostics, Germany) and samples prepared for western blot analysis were lysed with modified RIPA lysis buffer, while the lysis buffer for lung homogenate prepared for RT-qPCR analysis was used from the RNA extraction kit Nucleospin^®^ RNA II (Machery-Nagel, Germany), according to the manufacturer’s instructions. Total protein concentration was determined using BCA assay and total RNA concentration was measured with spectrophotometer (NanoDrop2000, Thermo Scientific) in the homogenised samples.

### Quantification of gene expression with RT-qPCR

Total RNA (1 μg) from each sample was reverse transcribed to cDNA using a Reverse Transcription-kit (PrimerDesign, UK) and quantitative PCR was performed using reagents from PrimerDesign. Subsequently, thermocycling and real-time detection of PCR products were performed on a sequence detection system (Stratagene, M×3000P, La Jolla, CA, USA) with standard cycling parameters. Genes of interest were calculated in relation to the reference gene 18S (PrimerDesign, UK) using the delta delta Ct method as previously described [11]. Gene expression was then normalised to its control sample at each time point (see Fig. 1). Data were presented with duplicate values as mean ± standard error of the mean (SEM). In Additional file 1: Table S1, supplementary data, primer sequences are shown for the following genes TNF-α, CCL2, IL-33, TSLP, RIG-I and TLR3 (from PrimerDesign, UK) and CCL5, IL-1β, IL-25, MDA5 and CCL11 (from Qiagen Sciences Inc, USA).

### Measured level of IL-33 protein in lung homogenates was determined by ELISA

IL-33 was analysed in lung homogenate using ELISA kit (R&D Systems, UK) according to the manufacturer’s instructions. Also total protein determination was performed from the same sample, and the results were then presented in relation to total protein concentration for each sample.

### Western blot analysis of PRR expression in lung homogenates

Western blot was performed for the quantification of PRR expression in lung. After total protein determination in lung homogenates, equal amount of each sample (20 μg protein) was loaded and electrophoresed on a 10 % polyacrylamide gel. The fractionated protein was blotted onto a PVDF membrane and blocked in blocking buffer (5 % non-fat dry milk, TBS-T). The membrane was then incubated in primary antibody; anti-RIG-I, anti-MDA5 or anti-TLR3. As loading control, an antibody was used directed towards the housekeeping protein β-tubulin. Appropriate secondary antibody was used directed towards each primary antibody, shown in Additional file 2: Table S2 as supplementary data. Proteins of interest were detected with detection kit SuperSignal^®^West Femto Maximum Sensitivity Substrate (Fischer Scientific AB, Sweden) and after exposure to chemiluminescence using LI-COR Odyssey Fc Imager system (LI-COR, Biosciences) and the software ImageStudio (2012 LI-COR Inc© version 3.1.4). The band density was calculated from the optical density and ratio was obtained after calculations in Microsoft Excel.

### Lung histology and immunostaining of IL-33 protein

Paraffin embedded lung tissues were sectioned 4 µm thick. Immunohistochemistry was used to visualise lung tissue IL-33 expression in paraffin embedded sections. The sections were first blocked with 5 % serum, following overnight incubation at 4 °C with primary goat anti-mouse IL-33 antibody (R&D Systems, UK). After rinsing the sections and incubating with secondary anti-goat IgG antibody (R&D Systems, UK), the staining was visualised with 3,3′-diaminobenzidine (DAB, Vectastain, Vector Laboratories, USA) and background stained with haematoxylin. All IL-33 stained sections were scanned (Aperio Technologies, USA) with software Aperio ScanScopeTM and representative photos were chosen and presented for each stimulus group in the study.

### Statistical analysis

Data are expressed as mean and SEM unless otherwise stated. The statistical test assessed in this study was unpaired t-test, after assumed Gaussian distribution. Kolmogorov–Smirnov test was performed to test normal distribution. p values of less than 0.05 were considered statistically significant. Statistics were performed with the software GraphPad Prism, version 6.0 (GraphPad Software, San Diego California USA, http://www.graphpad.com).

## Results

### Inflammatory cells in BALF at HDM-induced experimental asthma and during dsRNA-induced exacerbation phase

**Fig. 2:**
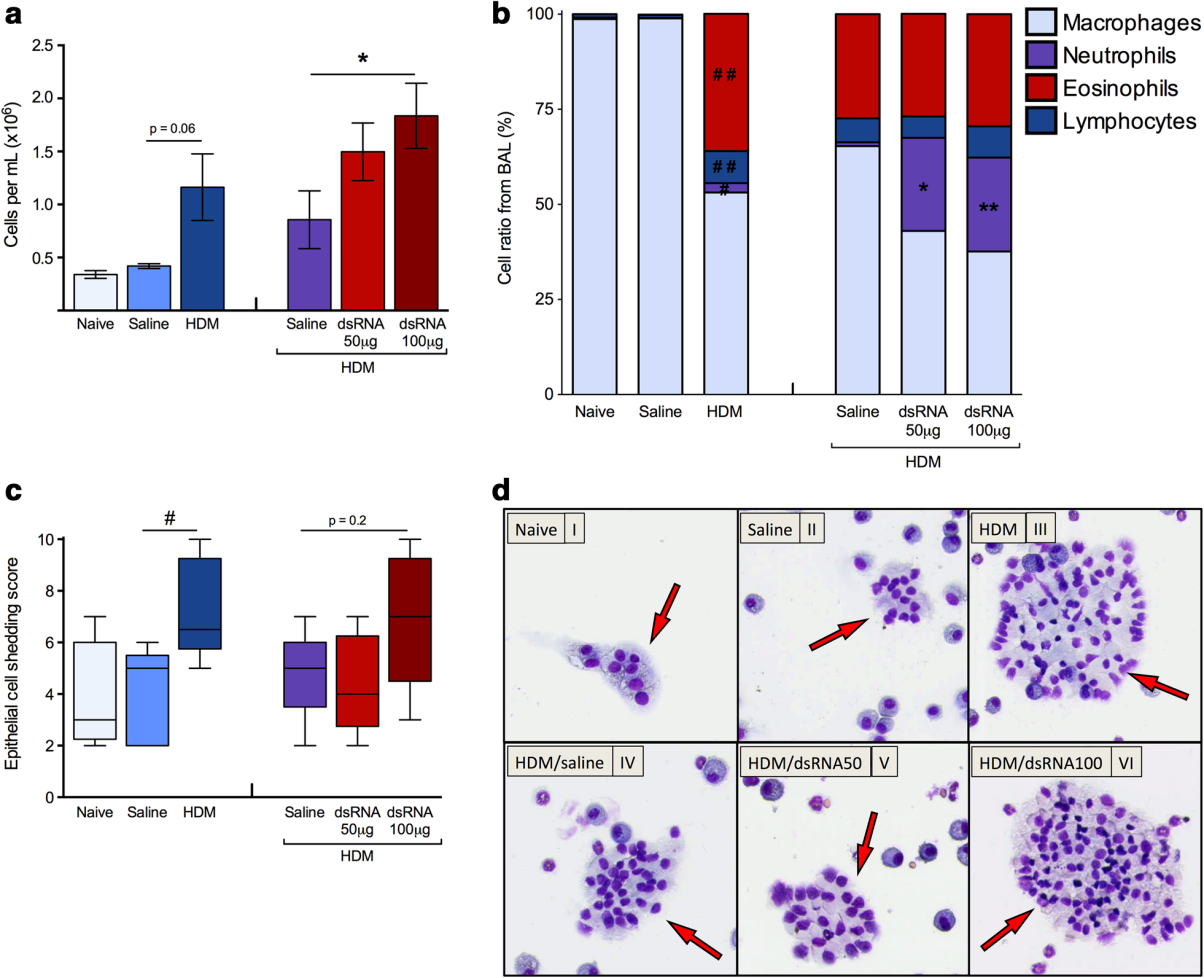
Effects of allergen and dsRNA administration on BALF cellular inflammation and indices of epithelial cell shedding. HDM allergen challenges alone increased total BALF cells (**a**) and increased eosinophils, neutrophils and lymphocytes compared to the non-allergic saline exposed group (**b**). At the exacerbation phase, in mice receiving additional dsRNA challenges, the total amount of cells in BALF was significantly- and dose-dependently increased by dsRNA compared to HDM/saline control (**a**, **b**). BALF neutrophils were much further enhanced by dsRNA and differential counts of eosinophils and lymphocytes were well sustained. The data are presented as mean ± SEM (n = 4–6 mice in each group). Simultaneously, epithelial cell shedding was estimated (**c**). BALF epithelial cell score was transiently increased after HDM challenge (**c**) and again tended to increase during the exacerbation phase (**c**). The data are presented as a *box* and *whisker plot*, shown with median and interquartile range with 95 % confidence intervals. Representative photos of the different groups from the study are shown (**d**), with *arrows* pointing out clusters of epithelial cells found in BALF (**d**). ^##^p < 0.01; ^#^p < 0.05 compared to saline control, while **p < 0.01; *p < 0.05 compared to HDM/saline control

Intranasal challenges with HDM were given for 3 weeks to induce experimental asthma in mice (Fig. 1) as previously described [22, 23]. By this regimen we recorded a threefold mean increase of total cells in BALF compared to saline-challenged control mice (p < 0.06; Fig. 2a). In agreement with the report by Gregory et al. [23] the differential cell count analysis showed that HDM succesfully induced macrophage-, lymphocyte-, neutrophil- as well as eosinophilic inflammation (Fig. 2b). Additionally, we demonstrated that the HDM-induced increase in mixed granulocytes is sustained. In mice with established HDM-induced experimental asthma receiving challenges with dsRNA to evoke exacerbation, BALF total cells were dose-dependently increased (Fig. 2a). dsRNA-induced exacerbations thus produced further graded increase in eosinophils, neutrophils, and lymphocytes above that induced by HDM alone (Fig. 2b and Additional file 3: Figure S1).

As indicated by bronchial epithelial cells present in BALF we discovered that HDM challenges alone produced airway epithelial cell shedding (Fig. 2c). This level of shedding was maintained at exacerbation caused by the high-dose of dsRNA (Fig. 2c, d).

### BALF total protein, cell necrosis marker and ATP in HDM-induced experimental asthma and during dsRNA-induced exacerbation phase

**Fig. 3:**
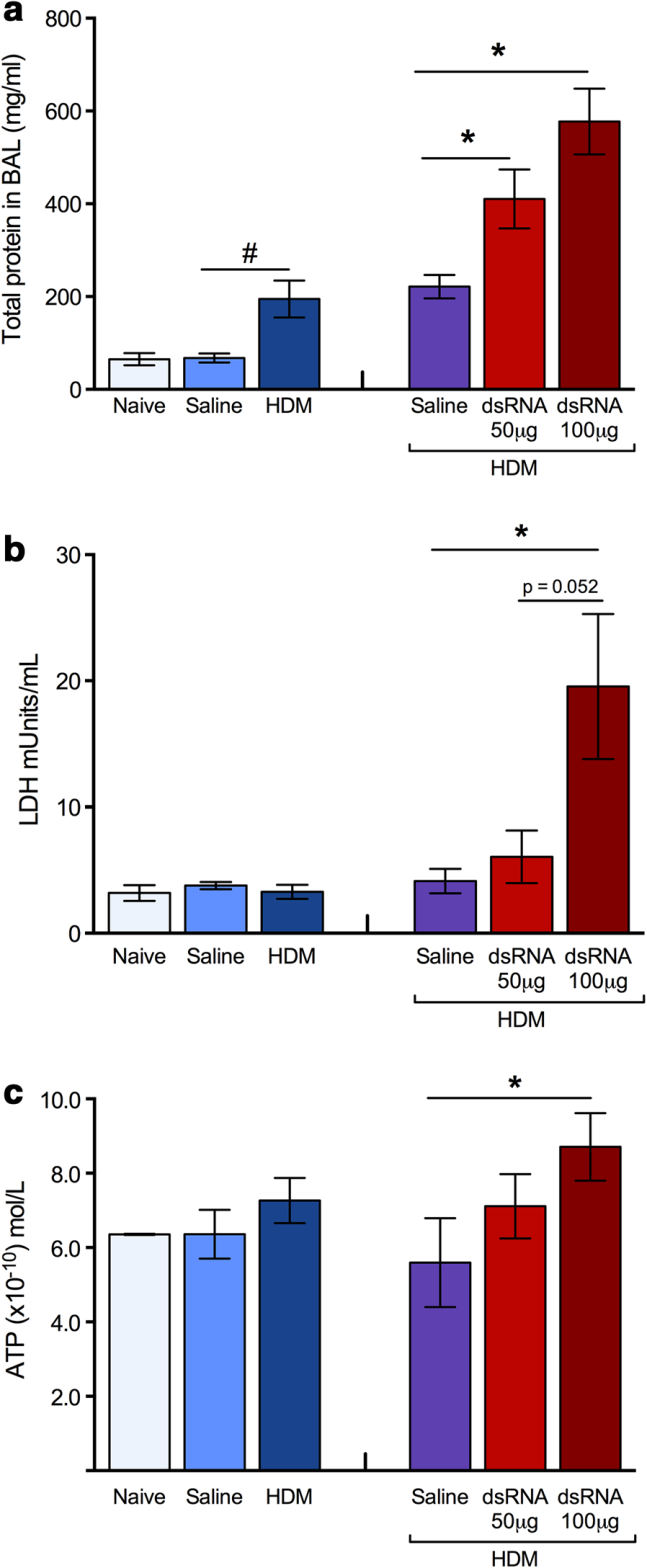
Exacerbation of airway inflammation induced by dsRNA in mice with established experimental HDM-asthma. BALF total protein levels increased after allergen challenge alone (**a**, *left*). This increase was sustained during additional saline administration, and was further dose-dependently enhanced at exacerbation by dsRNA (**a**, *right*). The cell necrosis biomarker LDH (**b**) and the alarmin ATP (**c**) were increased in BALF only at exacerbation by the high-dose of dsRNA. The data are presented as mean ± SEM (n = 4–6 mice in each group), ^#^p < 0.05 compared to saline control, while *p < 0.05 compared to HDM/saline control

We measured the total protein in BALF as a reflection of the general degree of ongoing inflammation in the lungs [6, 11]. Three weeks of intranasal allergen challenge increased total protein which was sustained. This inflammatory response was further dose-dependently enhanced by dsRNA (Fig. 3a). The cell necrosis marker LDH, previously shown to be induced in human rhinoviral-induced asthma exacerbations [20], was increased by the highest dose of dsRNA compared to saline control (Fig. 3b). HDM challenge alone did not increase LDH (Fig. 3b). ATP, which is considered an extracellular alarmin in asthma [21], was increased only in mice receiving the highest dose of dsRNA (Fig. 3c).

### dsRNA-induced exacerbation of experimental asthma augments lung tissue cytokines

**Fig. 4:**
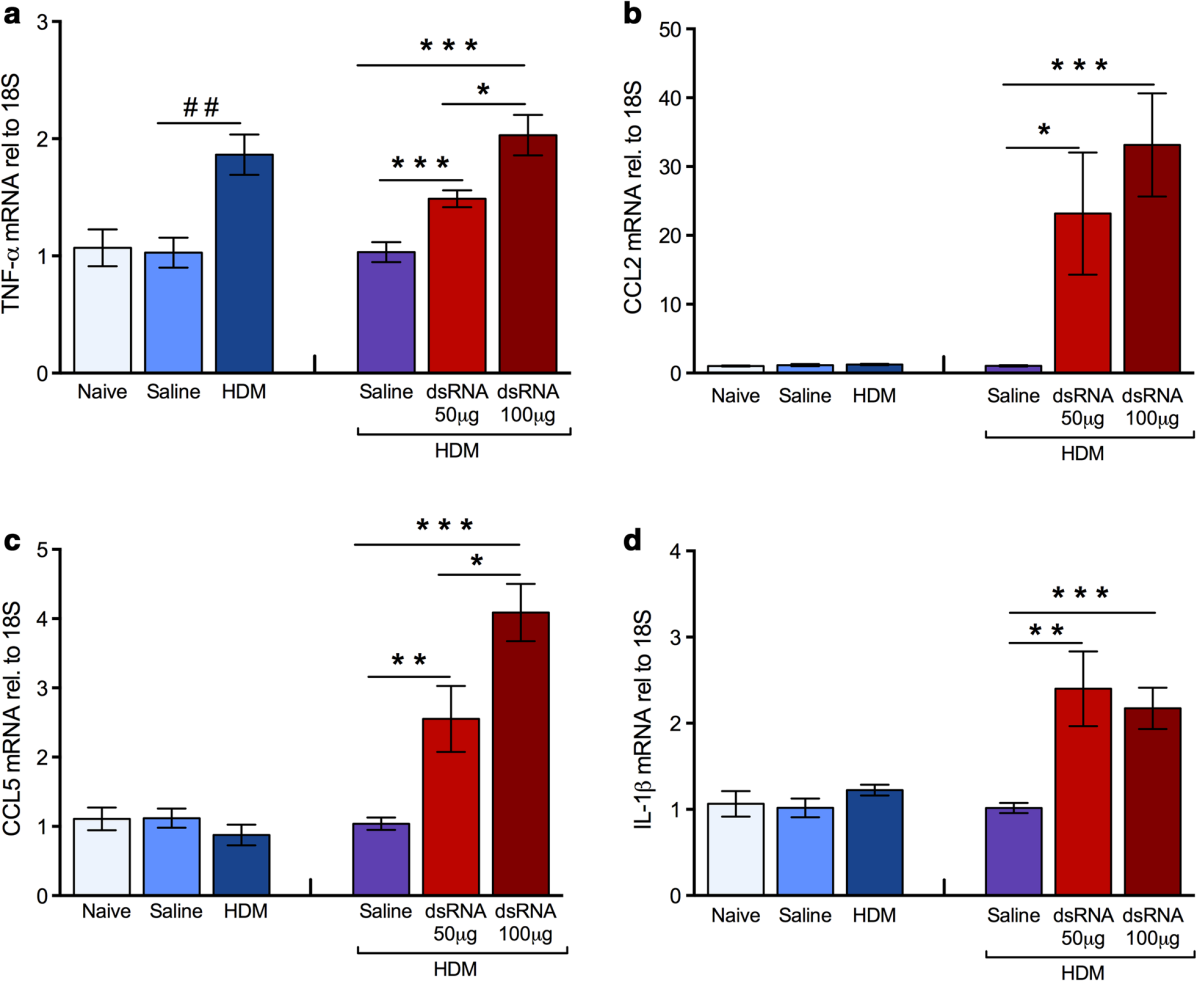
Inflammatory cytokines induced by HDM challenge and dsRNA exacerbation, respectively, in lung tissue. HDM challenge alone caused a transiently increased expression of only TNF-α (**a**) whereas exacerbation induced by additional dsRNA challenges increased the mRNA expression of all four cytokines; TNF-α (**a**) CCL2 (**b**), CCL5 (**c**) and IL-1β (**d**). The data are presented as mean ± SEM (n = 4–6 mice in each group). ^##^p < 0.01 compared to saline control, while ***p < 0.001; **p < 0.01; *p < 0.05 compared to HDM/saline control

Pro-inflammatory cytokines may play a causative role in asthma exacerbations [24, 25]. We therefore investigated the gene expression of four inflammatory cytokines in lung tissue; TNF-α, CCL2, CCL5 and IL-1β. Increased expression of all four cytokines was observed during exacerbation (Fig. 4a–d), whereas only TNF-α was significantly increased after HDM challenge alone compared to animals receiving saline challenge (Fig. 4d). In addition, gene expression of CCL11 was increased at dsRNA-induced exacerbation (Additional file 4: Figure S2).

### Gene expression of upstream TH_2_-type cytokines IL-33, TSLP and IL-25 at HDM-induced experimental asthma and during dsRNA-induced exacerbation phase

**Fig. 5:**
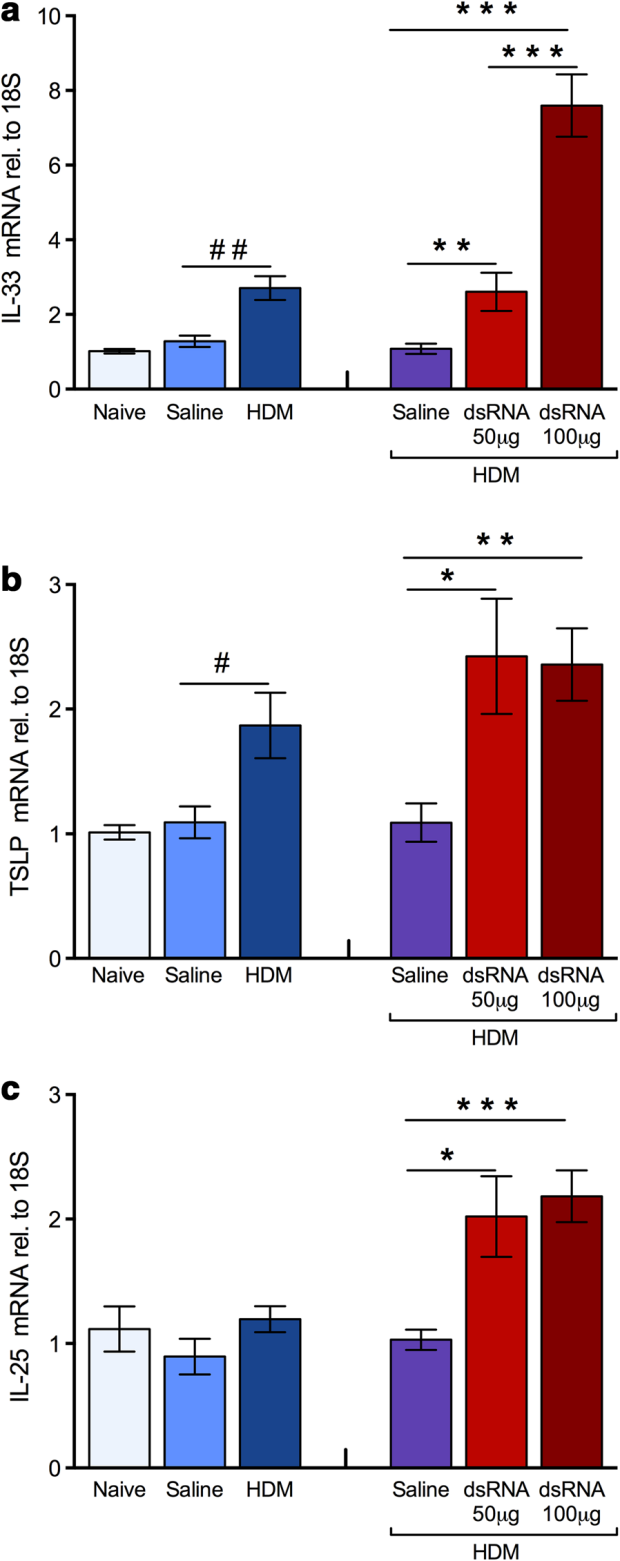
Upstream TH_2_-cytokines, IL-33, TSLP and IL-25 were induced at exacerbation. HDM challenges alone caused transiently increased lung tissue mRNA expression of IL-33 (**a**, *left*) and TSLP (**b**, *left*). dsRNA-induced exacerbation caused further increase in IL-33 (**a**, *right*) and TSLP (**b**, *right*). However, IL-25 mRNA expression was exclusively induced at exacerbation (**c**). The data are presented as mean ± SEM (n = 4–6 mice in each group). ^##^p < 0.01, ^#^p < 0.05 compared to saline control, while ***p < 0.001; **p < 0.01; *p < 0.05 compared to HDM/saline control

Three bronchial epithelial upstream cytokines; IL-33, TSLP and IL-25 are considered to trigger type-2 inflammation in asthma [1, 14, 26, 27]. IL-33 and TSLP, but not IL-25, were significantly induced by HDM challenge alone (Fig. 5a). However, the exacerbation caused by dsRNA induced markedly increased gene expression of all three cytokines; IL-33, TSLP and IL-25 (Fig. 5a–c).

### Lung tissue levels and distribution of IL-33 at HDM-induced experimental asthma and during dsRNA-induced exacerbation phase

**Fig. 6:**
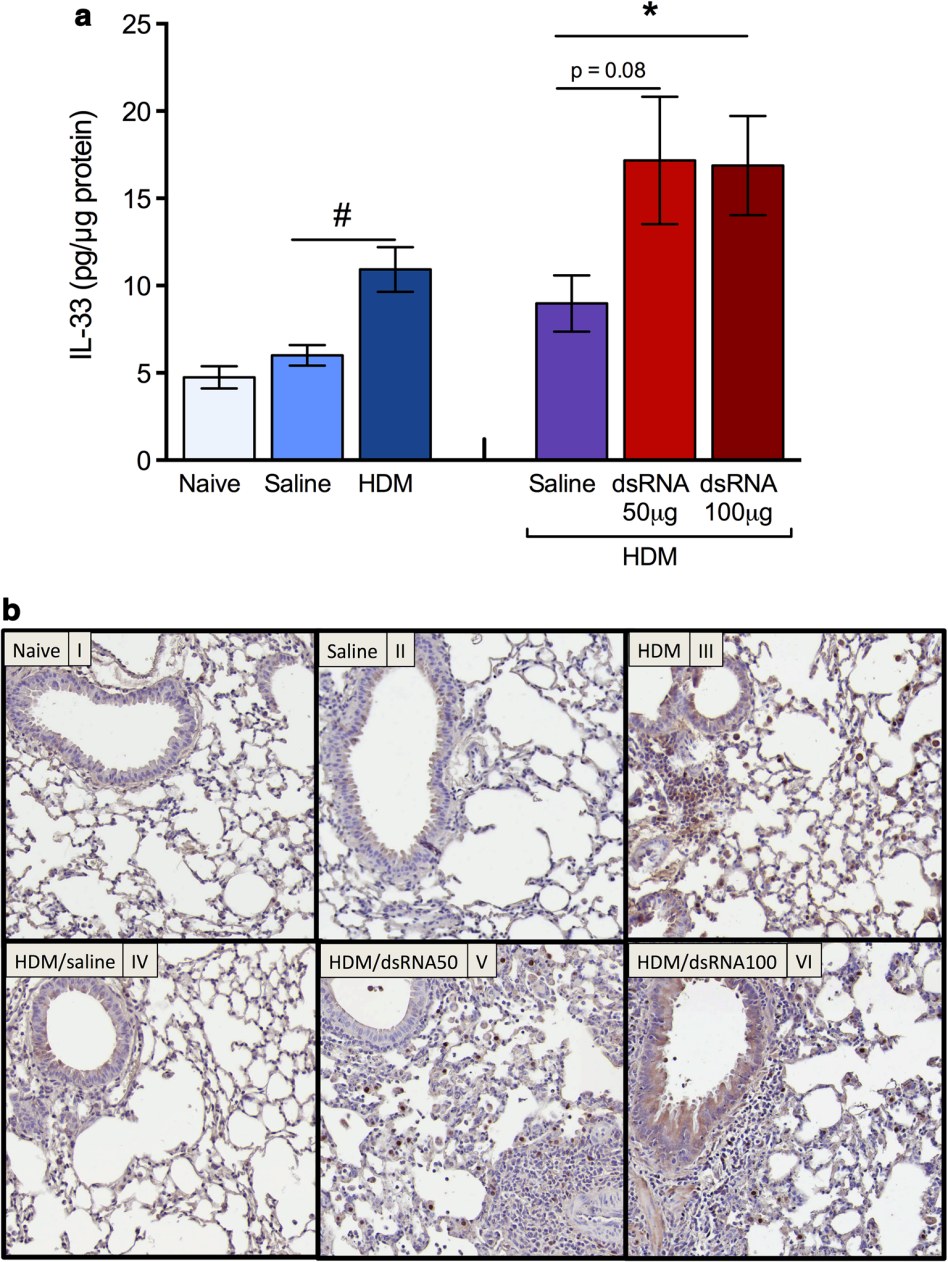
HDM-induced IL-33 protein was further increased at dsRNA-induced exacerbation. IL-33 protein in lung homogenate increased efter HDM challenge alone and was further increased by dsRNA-induced exacerbation (**a**). Immunohistochemistry showed IL-33 protein tissue occurrence in paraffin sections of the mouse lungs (**b**) in accord with the protein analysis (representative photos are shown). The data are presented as mean ± SEM (n = 4–6 mice in each group). ^#^p < 0.05 compared to saline control, while *p < 0.05 compared to HDM/saline control

Lung tissue IL-33 protein was increased by HDM challenge alone and further increased at exacerbation (Fig. 6a). Immunostaining of lung tissue sections showed IL-33 in bronchial epithelial cells, macrophages and in the smooth muscle cell layer, particularly in the exacerbation groups (Fig. 6b).

### Increased expression of lung pattern recognition receptors (PRRs) RIG-I, MDA5, and TLR3 only at exacerbation

**Fig. 7:**
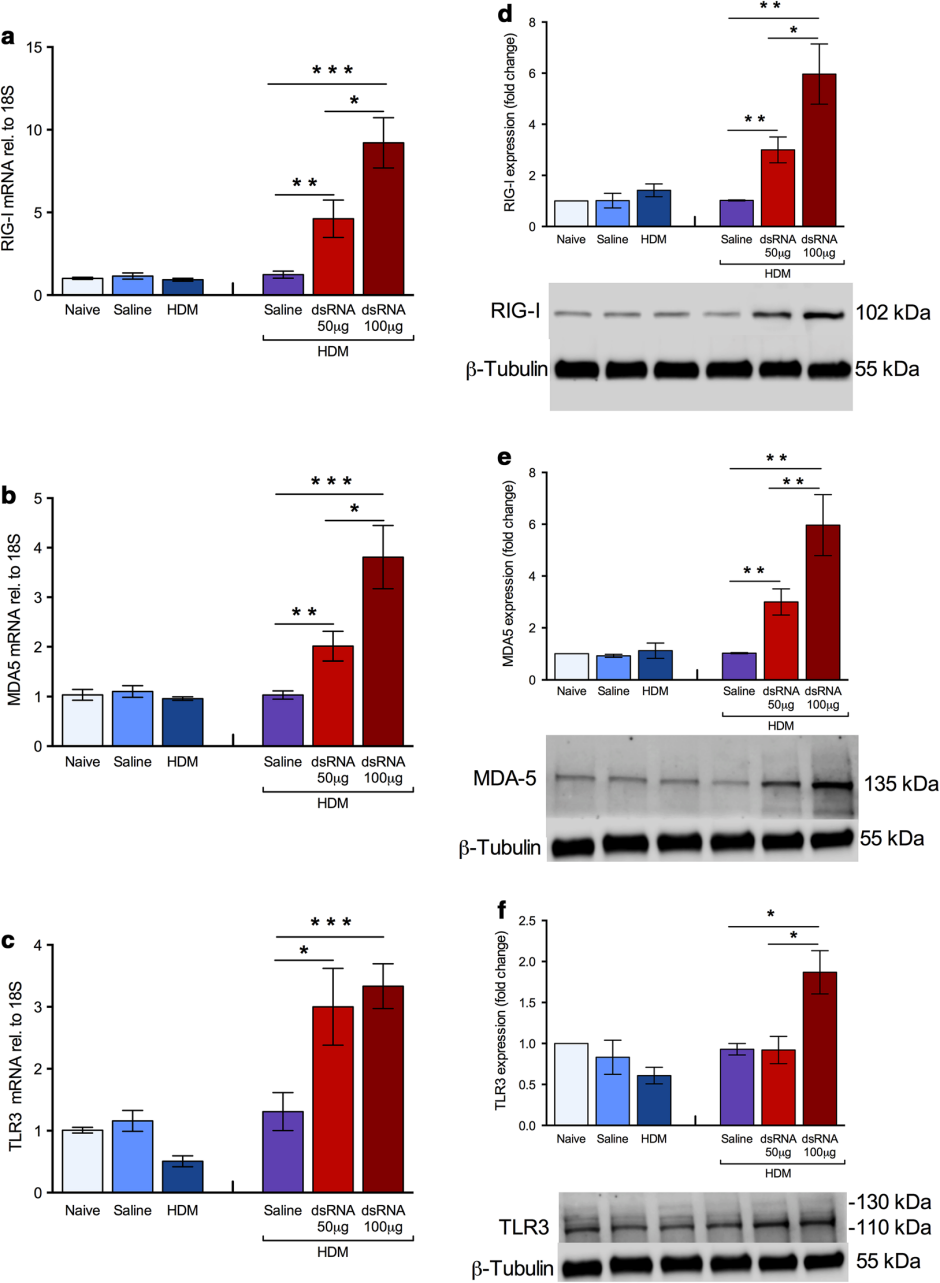
PRRs mRNA expression and protein levels were induced at exacerbation only. RIG-I (**a**), MDA5 (**b**), and TLR3 (**c**) mRNA were increased exclusively at dsRNA-induced exacerbation. Similarly, protein levels of RIG-I (**d**), MDA5 (**e**) and TLR3 (**f**) were also increased exclusively at exacerbations. Optical density was determined from western blot bands and related to housekeeping protein β-tubulin. Representative blots are shown below the calculated protein levels. The data are presented as mean ± SEM (n = 4–6 mice in each group), ***p < 0.001; **p < 0.01; *p < 0.05 compared to HDM/saline control

RIG-I, MDA5 and TLR3 gene expressions and protein were analysed using RT-qPCR and western blot, respectively. All PRRs were dose-dependently increased at dsRNA-induced exacerbation but were not altered by HDM challenge alone (Fig. 7a–f).

## Discussion

In this study we demonstrated that HDM-induced experimental asthma involved induction of lung tissue gene expression of four inflammatory cytokines as well as two upstream TH_2_- cytokines, IL-33 and TSLP. BALF data further indicated that the present HDM model was characterised by a sustained eosinophilic inflammation involving protein exudation, cell necrosis and recruitment of neutrophils and lymphocytes. These basic inflammatory cytokine- and cellular features of experimental asthma at baseline were dose-dependently exaggerated by the addition of a viral exacerbation stimulus, dsRNA. Furthermore, the PRRs; RIG-I, MDA5 and TLR3, as well as the cytokine IL-25 were robustly induced at exacerbation only, by dsRNA but not by HDM exposures alone. Our HDM-dsRNA model of experimental asthma exacerbation involves pulmonary inflammatory effects that agree with observations in human severe asthma.

In most types of asthma, bronchial eosinophilia is a central feature. Merely by local airway exposure, HDM produced allergic inflammation in this study that was clearly eosinophilic in nature. These present findings appear to be at variance with a previous report by Phan et al. [18] in which neither HDM challenges alone nor viral induced exacerbation was associated with pulmonary eosinophilia. The differing outcomes may reflect either different batches of HDM or different exposure regimens; Phan and colleges administered ten consecutive daily HDM challenges, whereas we administered HDM three times a week for three weeks. A mixed eosinophilic-neutrophilic condition is also a frequent observation in human asthma, especially in severe conditions. Hence, it is of note that we recorded a degree of neutrophilia along with a prominent eosinophilia already at the baseline asthma evoked by HDM challenges alone. Differing from previous data obtained using sensitisation and challenge with OVA [11, 12, 14, 28], the present HDM exposure alone was sufficient to increase lung expression of IL-33 and TSLP. Future studies seem warranted to determine possible roles of these immunoregulatory cytokines in sustained HDM-induced lung inflammation. By producing sustained inflammation, characterised by eosinophilia and protein exudation (measured here as ‘total protein’) several days after the final exposure to HDM, the present allergic model may compare favourably with common OVA-models of asthma where rather more transient eosinophilic inflammation has been observed [11, 12, 28]. Importantly, the baseline allergic lung inflammation in this study persisted into the period when exacerbation is induced by the additional dsRNA challenge.

dsRNA, or rather its synthetic mimic, Poly(I:C), is an archetypical TLR3 ligand increasingly employed as a rhinoviral infection surrogate in experimental studies in vitro and in vivo [11, 29–32]. In exacerbation models based on OVA-induced allergic inflammation additional exposures to dsRNA have increased BALF neutrophils but produced little increase in- or may even have reduced the numbers of eosinophils and lymphocytes [11, 33]. In this study, however, besides producing marked neutrophilia, dsRNA dose-dependently increased both eosinophils and lymphocytes above the baseline inflammation produced by HDM. This observation is of interest in view of the cellular features of asthma exacerbations that include markedly increased numbers of mixed granulocytes and lymphocytes [12, 28, 34–36]. It is possible that the increased expression of CCL11 induced by the present dsRNA-induced exacerbation contributed to the increased BALF eosinophilia. The mRNA expression of pro-inflammatory cytokines measured in this study; CCL2, CCL5 and IL-1β were only induced at exacerbation. This is in contrast to TNF-α being upregulated both after allergen challenge and at exacerbation. TNF-α can be one of the factors contributing to the increased neutrophil chemotaxis observed after allergen challenge alone and at exacerbation.

Gross aspects of severe asthma, beyond increased numbers of inflammatory cells, include protein exudation and occurrence of cell necrosis [6, 16, 20]. Hence, it is of interest that both total protein and a marker of cell necrosis, LDH, were much increased in the present study at exacerbation, the latter being increased exclusively at exacerbation. In 2002 Wark et al. demonstrated that BALF levels of LDH were increased at exacerbations of human asthma caused by viral infections [20]. However, few details are as yet known regarding what cell types become necrotic and release LDH in human asthma and in our model of exacerbation. The bronchial epithelium is a major target for rhinoviral infection in the airways [37, 38]. Infected and dying bronchial epithelial cells would therefore release LDH in the airways. Airway tissue eosinophils in severe asthma are ultimately activated by mechanisms leading to regulated necrosis of the eosinophils themselves, which may contribute to LDH levels. Further, epithelial cells have been severely deranged in proximity of the necrotic eosinophils and their released toxic proteins [16]. However, so far these aspects of exacerbations have not been studied well in animal models. Any cell undergoing necrosis may release danger associated molecular patterns (DAMPs). It is possible that the present increase in ATP, a known alarmin and DAMP in asthma [21, 39], reflects in part necrosis effects. Also IL-33, which was significantly increased in this study, may be released as an alarmin at cell death [40]. In addition, IL-33 has been demonstrated to be released by non-necrotic mechanisms [41]. PRRs (TLR3, MDA5, RIG-I) that sense virus-derived RNA, or dsRNA used in this study, exert a variety of anti-viral immune responses. Recent reports suggest that these effects include PRR-induced cell necrosis [42, 43]. Hence, it is possible that the increased expression of PRRs, which we observed exclusively at the dsRNA-induced exacerbations, may be involved in cell necrosis mechanisms during the present exacerbations. We suggest that our exacerbation model involving increased LDH levels may be suited for further studies of cells and factors associated with inflammatory necrosis in asthma.

Neither OVA nor HDM models of asthma have been characterised by epithelial shedding [44]. Yet, epithelial shedding is a hallmark of asthma. It is therefore a potentially important observation that HDM challenge alone was sufficient to produce epithelial shedding in this study. Furthermore, the shedding produced clusters of epithelial cells reminiscent of Creola bodies found in asthmatic sputa, especially at exacerbations [45]. Shed epithelial cells are removed by airway clearance mechanisms and there is no such de novo recruitment of epithelial cells as occurs with inflammatory cells. Hence, specifically designed in vivo studies are warranted to examine in detail any significance of the maintained level of epithelial shedding at the point of measurement at exacerbation in this study. Epithelial shedding-regeneration is by itself a pro-inflammatory and pro-remodelling process that could contribute to the cellular and protein airway features of severe asthma and would therefore be a warranted component of future models of asthma [16, 44].

In general, the present asthma exacerbation was characterised by pronounced features of inflammation already at a low challenge dose of dsRNA (50 μg). Indeed, on important variables such as TSLP and IL-25, a maximal or near maximal effect was produced by the 50 μg dose regimen. Hence, it may be difficult to compare the present model with previous reports where up to 200 μg dose of dsRNA has been employed [12, 13]. Furthermore, several of the present BALF analyses or lung tissue gene expressions may not have been determined in the prior art involving dsRNA-induced challenges on top of the HDM-challenge. In this study we were particularly interested to see significantly increased mRNA expression of IL-33, TSLP and IL-25. This epithelial-derived troika is now considered as major upstream cytokines regulating TH_2_-induced inflammation [1]. By the rate that novel effects of these cytokines are being discovered it seems increasingly likely that they may be causally involved in inflammatory processes also beyond the TH_2_-type responses. The present increased IL-33 expression was also evident by increased IL-33 protein particularly at the exacerbations. Exacerbation studies involving OVA-induced inflammation and viral infection indicated that IL-25 is importantly involved [14]. Future studies are warranted to define possible roles of IL-33, TSLP and IL-25 in the present HDM-based dsRNA exacerbation model. However, translation of actual roles of inflammatory mechanisms in pathogenesis of asthma from mouse to human is notoriously difficult [15, 44, 46]. Yet pharmacological interventions that reduce the induction of cytokines in in vivo animal models may retain this property in humans. To this end the present model may serve well for in vivo drug discovery studies aiming at regulation of lung tissue expression of IL-33, TSLP and IL-25 for possible therapeutic potential.

## Conclusion

In conclusion, our new model of viral-induced asthma exacerbation employing a combination of HDM- and dsRNA-challenges has shown translational value. Its asthma-like features include indices such as mixed granulocytic inflammation, epithelial shedding, protein exudation and cell necrosis. Our data suggests further that the present model should be particularly useful for in vivo studies involving pharmacological effects on exacerbation-induced expression of the three upstream TH_2_-cytokines; IL-33, TSLP and IL-25, as well as PRRs.

## Additional files

10.1186/s12967-016-0808-x Commercially available primer sequences for genes that were analysed with RT-qPCR.
10.1186/s12967-016-0808-x Primary and secondary antibodies used during western blot analysis of lung homogenate samples. All primary antibodies were diluted in TBS-T with 5 % BSA, while the secondary antibody was diluted in TBS-T with 5 % milk.
10.1186/s12967-016-0808-x Increased number of eosinophils, neutrophils, and lymphocytes in BALF at exacerbation. From the total- and differential cell count, the total number of immune cells was obtained. Here showing the total amount of eosinophils (A), neutrophils (B) and lymphocytes (C), all being dose-dependently induced at exacerbation, compared to Saline-control. The data are presented as mean ± SEM (n = 5–6 mice in each group), ** p < 0.01; * p < 0.05 compared to HDM/saline control.
10.1186/s12967-016-0808-x Eosinophil chemotactic CCL11 (Eotaxin) was dose dependently induced at exacerbation. The mRNA expression of CCL11 was dose-dependently increased at exacerbation. The data are presented as mean ± SEM with individual values in duplicates and n = 5–6 mice in each group. ** p < 0.001; * p < 0.05 compared to HDM/Saline control.
