# Supplementary material for: Increased expression of upstream TH2-cytokines in a mouse model of viral-induced asthma exacerbation
Source: J Transl Med. 2016 Feb 16;14:52. doi: 10.1186/s12967-016-0808-x (PMC4754855; doi:10.1186/s12967-016-0808-x)
Supplement: Supplementary file 1 — 10.1186/s12967-016-0808-x Commercially available primer sequences for genes that were analysed with RT-qPCR. [file 12967_2016_808_MOESM1_ESM.docx]

Additional file 1:

**Table S1**. Commercially available primer sequences for genes that were analysed with RT-qPCR.

**Gene Source Sequence**

TNF-α: PrimerDesign AGCCAGGAGGGAGAACAGA (forward)

CAGTGAGTGAAAGGGACAGAAC (reverse)

CCL2: PrimerDesign TGAAGTTGACCCGTAAATCTGAA (forward)

AGGCATCACAGTCCGAGTC (reverse)

IL-33: PrimerDesing CAATGTTGACGACTCTGGAAAAG (forward)

GGGACTCATGTTCACCATCAG (reverse)

TSLP: PrimerDesign AAACTGAGAGAAATGACGGTACT (forward)

TCTGGAGATTGCATGAAGGAATA (reverse)

RIG-I: PrimerDesign CGATATTTTGAAAGACTTGGGTACA (forward)

ATGGCTCCGTTGTTGAGATTG (reverse)

TLR3: PrimerDesign AAGTTATTCGCCCTCCTCTTGA (forward)

AGATTCTGGATGCTTGTGTTTGA (reverse)

CCL5: QIAGEN Mm_Ccl5_2_SG (QT01747165) QuantiTect Primer Assay, 107bp

IL-1β: QIAGEN Mm_Il1b_2_SG (QT01048355) QuantiTect Primer Assay, 150bp

IL-25: QIAGEN Mm_Il25_1_SG (QT00134645) QuantiTect Primer Assay, 148bp

MDA-5: QIAGEN Mm_Ifih1_1_SG (QT00156338) QuantiTect Primer Assay, 118bp

CCL11: QIAGEN Mm_Ccl11_1_SG (QT00114275) QuantiTect Primer Assay,109bp
