# Supplementary material for: Increased expression of upstream TH2-cytokines in a mouse model of viral-induced asthma exacerbation
Source: J Transl Med. 2016 Feb 16;14:52. doi: 10.1186/s12967-016-0808-x (PMC4754855; doi:10.1186/s12967-016-0808-x)
Supplement: Supplementary file 2 — 10.1186/s12967-016-0808-x Primary and secondary antibodies used during western blot analysis of lung homogenate samples. All primary antibodies were diluted in TBS-T with 5 % BSA, while the secondary antibody was diluted in TBS-T with 5 % milk. [file 12967_2016_808_MOESM2_ESM.docx]

Additional file 2:

**Table S2.** Primary and secondary antibodies used during western blot analysis of lung homogenate samples.

All primary antibodies were diluted in TBS-T with 5% BSA, while the secondary antibody was diluted in TBS-T with 5% milk.

**Primary Antibody made in dilution source**

RIG-I: rabbit 1:1000 Cell Signaling; Danvers, MA, USA

MDA5 rabbit 1:1000 Cell Signaling; Danvers, MA, USA

TLR3: rabbit 1:1000 Cell Signaling; Danvers, MA, USA

β-Tubulin rabbit 1:1000 Cell Signaling; Danvers, MA, USA

**Secondary Antibody made in dilution source**

anti-rabbit IgG goat 1:3000 Fischer Scientific AB Gothenburg, Sweden
