# Supplementary figures and images for: Increased expression of upstream TH2-cytokines in a mouse model of viral-induced asthma exacerbation
Source: J Transl Med. 2016 Feb 16;14:52. doi: 10.1186/s12967-016-0808-x (PMC4754855; doi:10.1186/s12967-016-0808-x)

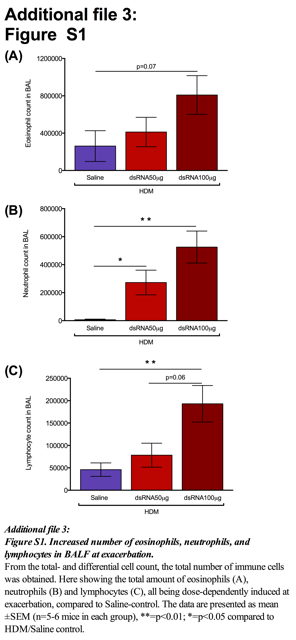

Supplement: Supplementary file 3 — 10.1186/s12967-016-0808-x Increased number of eosinophils, neutrophils, and lymphocytes in BALF at exacerbation. From the total- and differential cell count, the total number of immune cells was obtained. Here showing the total amount of eosinophils (A), neutrophils (B) and lymphocytes (C), all being dose-dependently induced at exacerbation, compared to Saline-control. The data are presented as mean ± SEM (n = 5–6 mice in each group), ** p < 0.01; * p < 0.05 compared to HDM/saline control. [file 12967_2016_808_MOESM3_ESM.tiff]

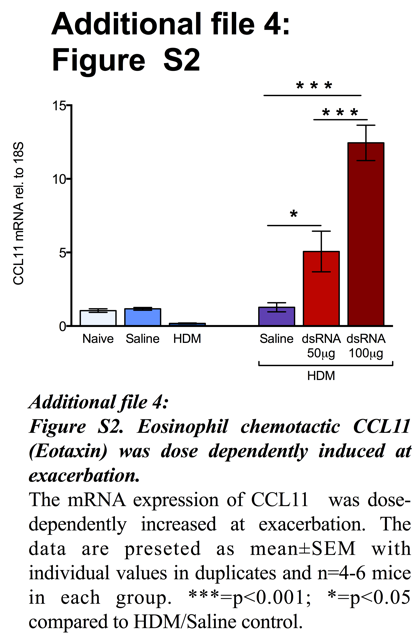

Supplement: Supplementary file 4 — 10.1186/s12967-016-0808-x Eosinophil chemotactic CCL11 (Eotaxin) was dose dependently induced at exacerbation. The mRNA expression of CCL11 was dose-dependently increased at exacerbation. The data are presented as mean ± SEM with individual values in duplicates and n = 5–6 mice in each group. ** p < 0.001; * p < 0.05 compared to HDM/Saline control. [file 12967_2016_808_MOESM4_ESM.tiff]
